# Computational approaches to interpreting genomic sequence variation

**DOI:** 10.1186/s13073-014-0087-1

**Published:** 2014-10-22

**Authors:** Graham RS Ritchie, Paul Flicek

**Affiliations:** European Molecular Biology Laboratory, European Bioinformatics Institute, Hinxton, Cambridge CB10 1SD UK; Wellcome Trust Sanger Institute, Hinxton, Cambridge CB10 1SA UK

## Abstract

Identifying sequence variants that play a mechanistic role in human disease and other phenotypes is a fundamental goal in human genetics and will be important in translating the results of variation studies. Experimental validation to confirm that a variant causes the biochemical changes responsible for a given disease or phenotype is considered the gold standard, but this cannot currently be applied to the 3 million or so variants expected in an individual genome. This has prompted the development of a wide variety of computational approaches that use several different sources of information to identify functional variation. Here, we review and assess the limitations of computational techniques for categorizing variants according to functional classes, prioritizing variants for experimental follow-up and generating hypotheses about the possible molecular mechanisms to inform downstream experiments. We discuss the main current bioinformatics approaches to identifying functional variation, including widely used algorithms for coding variation such as SIFT and PolyPhen and also novel techniques for interpreting variation across the genome.

## The need for variant annotation

Modern genomics technologies are yielding extensive catalogues of sequence variation. Substantial progress has been made in identifying some of the genetic contribution to disease, but for many of the genotype-phenotype associations discovered, we do not yet understand the molecular mechanisms by which the underlying sequence variants are acting. To make sense of this vast amount of data in a timely manner, high-throughput techniques are required to filter and prioritize candidate variants on the basis of the wide range of functional genomic data that are currently available. Numerous computational approaches have been developed and applied in the search for sequence variants that play a role in phenotypes of interest. These methods vary substantially in their underlying algorithmic approaches, and these differences lead to a number of considerations that should be taken into account when interpreting the results. This article discusses a number of widely used approaches to variant annotation, which we categorize according to the underlying algorithmic strategy. For each category, we discuss some of the advantages and limitations of the approach.

We first examine tools that identify overlaps with annotated functional genomic regions, such as genes and regulatory elements, and software that leverages existing biological knowledge to predict the effects of sequence variation in these regions. A number of methods have also been developed that use signatures of evolutionary constraint to identify conserved regions where variation is likely to be deleterious. Machine-learning techniques that integrate diverse sources of information to predict likely functional variants have also been widely applied to interpret variation in coding regions, and recently also variation in the non-coding regions of the genome. Finally, several new methods aimed at discovering novel trait associations that can incorporate functional information are described. Although we have not attempted to be exhaustive, all of the tools discussed, along with relevant URLs and references, are listed in Table 1.

**Table 1:** **A summary of selected computational tools and their applications**

**Tool**	**Application**	**Comments**	**URL**	**Reference**
***Annotation based on overlap with and proximity to functional elements***				
Ensembl Genome Browser	Manual variant annotation and genomic context	Web server, data also available via Perl and REST APIs	http://www.ensembl.org	[10]
UCSC Genome Browser	Manual variant annotation and genomic context	Web server, data also available for download using the UCSC table browser	http://www.genome.ucsc.edu	[11]
Bedtools	Automatic high performance feature overlap and proximity	Command line tool and Python interface	http://bedtools.readthedocs.org	[12]
Bedops	Automatic high performance feature overlap and proximity	Command line tool	http://bedops.readthedocs.org	[13]
HaploReg	Web server identifying non-coding annotations for variants and haplotypes	Web server with pre-computed results for several GWAS	http://www.broadinstitute.org/mammals/haploreg/	[14]
***Biologically informed rule-based annotation***				
Ensembl Variant Effect Predictor (VEP)	Wide support for variant annotation, emphasis on genic variants, but also incorporates regulatory elements and TF motifs from JASPAR	Downloadable software, web server, Perl and REST APIs, plugin system to add functionality	http://www.ensembl.org/vep	[17]
ANNOVAR	Annotation of genic variants, can also identify overlaps with other annotated elements	Downloadable software	http://www.openbioinformatics.org/annovar/	[18]
VAT	Annotation of genic variants	Downloadable software	http://vat.gersteinlab.org	[20]
SnpEff	Annotation of genic variants, companion tool SnpSift can filter results by annotations	Downloadable software	http://snpeff.sourceforge.net	[19]
RegulomeDB	Identifies overlaps with non-coding elements and applies heuristic rules to predict consequences	Web server	http://regulome.stanford.edu	[24]
***Annotation based on sequence motifs***				
JASPAR	Open access database of TF binding PWMs	Queryable interface and database downloads	http://jaspar.genereg.net	[26]
MEME suite	Several tools for handling PWMs	Web services and downloadable tools	http://meme.nbcr.net	[27]
MOODS	Tool for aligning PWMs to sequences	Command line tool	http://www.cs.helsinki.fi/group/pssmfind/	[28]
Human Splicing Finder	Tool for computing the effects of mutations on splicing	Web server	http://www.umd.be/HSF/	[29]
***Annotation based on constraint estimated from multiple sequence alignments***				
GERP	Nucleotide resolution conservation scores	Downloadable software, pre-computed scores and elements for human and mouse genomes	http://mendel.stanford.edu/SidowLab/downloads/gerp/	[31]
PHAST package	Suite of tools for phylogenetic analyses, including phastCons and phyloP	Downloadable software and R package	http://compgen.bscb.cornell.edu/phast/	[32,33]
SCONE	Position-specific conservation scores	Downloadable software	http://genetics.bwh.harvard.edu/scone/	[34]
SIFT	Predicts deleterious AASs) based on conservation and physico-chemical principles	Downloadable software and web server	http://sift.bii.a-star.edu.sg	[35]
FATHMM	Uses a hidden Markov model to identify AASs likely to be deleterious	Downloadable software and web server, VEP plugin	http://fathmm.biocompute.org.uk	[39]
***Integrative approaches using supervised learning algorithms***				
PolyPhen	Predicts deleterious AASs based on several sequence and structural features	Downloadable software and web server, pre-computed predictions for all possible substitutions	http://genetics.bwh.harvard.edu/pph2/	[41]
MutationTaster	Classifier which can predict deleterious variants in genic regions, including coding regions and splice sites	Web server	http://www.mutationtaster.org	[42]
MutationAssessor	Predicts deleterious AASs based on evolutionary conservation	Web server, pre-computed scores for all possible substitutions	http://www.mutationassessor.org	[43]
SNAP	Predicts deleterious AASs based on a range of protein level information	Downloadable software and web server	http://www.rostlab.org/services/SNAP/	[44]
PhD-SNP	Predicts deleterious AASs based on protein sequence information	Downloadable software and web server	http://snps.biofold.org/phd-snp/	[45]
Condel	Tool that integrates predictions from multiple AAS prediction tools	Downloadable software and web server, VEP plugin	http://bg.upf.edu/fannsdb/	[46]
CAROL	Tool that integrates scores from SIFT and PolyPhen using a weighted Z method	Downloadable R script, VEP plugin	http://www.sanger.ac.uk/resources/software/carol/	[47]
GWAVA	Classifier identifying likely functional regulatory variants	Downloadable software and database of pre-computed scores and annotations for known variants, VEP plugin	http://www.sanger.ac.uk/resources/software/gwava/	[48]
CADD	Integrated classifier that can score all classes of variants	Web server, pre-computed scores for all possible SNVs, VEP plugin	http://cadd.gs.washington.edu	[51]
***Phenotype association techniques that can incorporate functional information***				
fgwas	Command line tool for incorporating functional information into a GWAS	Downloadable software	http://www.github.com/joepickrell/fgwas	[52]
SKAT	A test for association between a set of variants and dichotomous or quantitative phenotypes	Downloadable software	http://www.hsph.harvard.edu/skat/	[53]
VT	Tests for pooled association of multiple rare variants and phenotypes	Downloadable software	http://genetics.bwh.harvard.edu/vt/dokuwiki/start	[54]
VAAST	Probabilistic tool to identify causal genes and variants in disease	Downloadable software, free for academic use, license required for commercial usage	http://www.yandell-lab.org/software/vaast.html	[55,56]

*Abbreviations*: *AAS* amino acid substitution, *API* application programming interface, *GWAS* genome-wide association studies, *PWM* position weight matrix, *REST* representational state transfer (an architecture style for designing networked applications), *TF* transcription factor, *UCSC* University of California Santa Cruz, *VEP* Variant Effect Predictor.

## Approaches to annotation

### Annotation based on overlap with and proximity to functional elements

A great deal of recent work in genomics and molecular biology has yielded rich and detailed annotation of the genome. Projects such as GENCODE [1] and RefSeq [2] continue to provide comprehensive annotation of both protein-coding genes and several classes of non-coding transcripts. Genic variants have been implicated in a wide range of genetic diseases, including sickle-cell disease, phenylketonuria and cystic fibrosis [3]. The ENCODE [4] and Roadmap Epigenomics [5] projects have expanded annotation beyond genic regions and have made available a wide range of annotations of regulatory elements in a range of different cell and tissue types. These elements include regions of open chromatin, regions marked by a range of histone modifications identifying epigenetic states, and sequences bound by specific transcription factors. Variation in regulatory elements has historically received less attention than that in protein-encoding regions, but the fact that the majority of variants associated with complex disease are found outside of genes suggests that at least some associations may be driven by variants that affect gene regulation [6]. Indeed, several recent studies have implicated specific regulatory variants in human diseases, such as type 2 diabetes [7], systemic lupus erythematosus [8] and hemophilia [9].

When seeking to interpret sequence variation, one can exploit the fact that all of the elements, both genic and regulatory, that show variation are typically mapped to a common genome assembly. Hence it is possible to identify functional elements that are overlapping or proximal to mapped sequence variants. Where the numbers of variants being investigated are low, such analyses can be performed manually using genome browsers such as Ensembl [10] and UCSC [11] by querying for variants by database identifiers (such as dbSNP or refSNP IDs) or by genomic position. For larger analyses, automated approaches are clearly required. Toolkits such as bedtools [12] and bedops [13] implement efficient data structures and algorithms to carry out these analyses on a genome scale very quickly. Both packages take as input databases of genomic elements in standard file formats, and support a range of useful operations such as computing overlaps and differences between sets of elements and identifying proximal elements. The webserver HaploReg [14] can also identify overlaps of variants and a wide range of non-coding elements from the ENCODE and Roadmap Epigenomics projects.

The identification of overlapping annotations can give a sense of the genomic context of a variant, but it is also important to consider in which elements variation might be tolerated. Several recent studies using genome-wide variation data from different human populations have sought to identify informative annotations by looking at patterns of variation overlapping a range of annotated elements. Ward and Kellis [15] used variation data from the 1000 Genomes Project to demonstrate that a wide range of annotated elements, including non-coding regions, show evidence of purifying selection in the human lineage, and their results identify constrained regions where sequence variation might be expected to be deleterious. Maurano *et al*. [16] used data identifying regions of open chromatin from DNase-seq experiments in numerous cell types and tissues. They demonstrated that trait-associated variants from genome-wide association studies (GWAS) are systematically enriched in open chromatin in relevant cell types: for example, they identified a significant enrichment of variants associated with Crohn’s disease, an autoimmune disorder, in regions of open chromatin in immune cells. The results of these and similar studies can be used to identify classes of annotation that might be informative when studying the effects of variation for some specific phenotype.

### Biologically informed rule-based annotation

**Figure 1:**
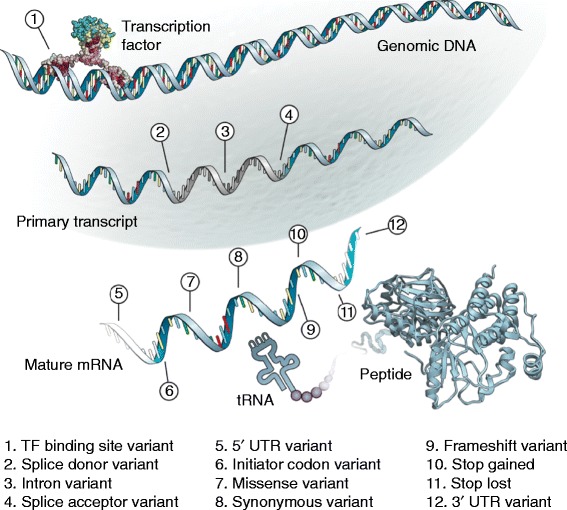
**A set of annotation terms used to describe the potential effects of sequence variants according to the genic regions they fall in and their allele sequences.** The terms are drawn from the Sequence Ontology and are depicted on the molecules they are predicted to affect. Variants categorized as any of the terms 2, 4, 9 and 10 are often collectively referred to as ‘loss-of-function’ variants, and are typically expected to severely affect gene function [25].

For some classes of genomic features, we have a relatively rich understanding of the function of particular nucleotide sequences, and this knowledge can be exploited to make allele-specific predictions about the effect of variants that overlap an element. For variants that fall within annotated gene structures, an understanding of the genetic code and splicing can be used to identify variants that change the coding sequence or disrupt the essential splice sites at either end of the intron (Figure 1). There are a number of software packages that perform these analyses, including the Ensembl Variant Effect Predictor (VEP) [17], ANNOVAR [18], SnpEff [19] and VAT [20]. As an example of the methodology implemented in these tools, the VEP starts with a predefined set of variant classifications (termed ‘consequences’), each of which has an associated rule for calling a consequence (which is expressed in the software). For example, the rule to call a frameshift variant is that a variant falls in coding sequence and that the absolute difference between the lengths of the reference and alternative alleles is not divisible by 3. The VEP systematically checks all rules against the query variants and outputs all consequence terms that hold for each variant. The VEP also reports ancillary information identified as part of the annotation process, such as predicted amino acid alterations and protein and cDNA relative coordinates, which could be useful in follow-up experiments.

Clearly, the predictions from such tools are heavily dependent on the gene set used, and it is important to realize that the human genome is still imperfectly annotated [21]. McCarthy and colleagues [22] have demonstrated that both the choice of gene set and software package can result in substantially different annotation results for the same set of query variants. (In their study, they compare the GENCODE and RefSeq gene sets, and the VEP and ANNOVAR packages). Their results suggest that there is still some ambiguity about how to assign consequence predictions to variants in some contexts, and efforts to define and standardize terms that are used to describe these effects, such as the Sequence Ontology [23], should help to improve the evaluation of different packages.

The importance of specific sub-sequences within other kinds of annotated regions, for example enhancer elements, is less well understood. Nevertheless, heuristic rules can still be productively applied to identify consistent combinations of annotations that are suggestive of possible function. The RegulomeDB [24] webserver identifies sequence variants that overlap with a wide range of data from the ENCODE and NIH Roadmap Epigenomics projects, transcription factor (TF) binding motifs and variants known to be associated with differences in gene expression (expression quantitative trait loci (eQTLs)). RegulomeDB uses all observed overlaps for a variant to assign it a score that is dependent on the consistency and specificity of the annotations. Thus, a variant overlapping a known eQTL, a TF motif and evidence for the binding of that specific TF (from a ChIP-seq experiment, for example) will be assigned a higher score than a variant that is only found to overlap a region of open chromatin.

Rule-based approaches are appealing in that they provide testable hypotheses regarding variant function, but they are of course limited by our current models of the function of genomic elements and so cannot identify variants acting in unexpected ways. Current implementations, especially those for genic variants, typically do not consider information about the relevant tissue or developmental stage in which the element might be important. For example, a variant that is predicted to terminate a coding sequence prematurely might have little effect if the relevant transcript is not expressed in a given tissue. Incorporating functional genomic data, such as expression levels in the tissue(s) of interest, with annotation results is therefore advised if possible. Population genetic data also indicate that some predicted ‘loss-of-function’ variants (Figure 1 legend) are also common in human populations: it has been predicted that a typical human is homozygous for approximately 20 such variants [25]. This perhaps surprising result suggests that not all variants that are predicted to truncate proteins have any significant phenotypic impact, and that we should be cautious in applying general rules about biological function across the genome.

### Annotation based on sequence motifs

**Figure 2:**
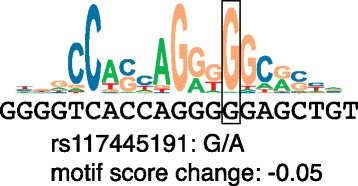
**A sequence logo for the transcriptional factor CTCF derived from binding site predictions from Ensembl on human chromosome 22.** The height of the letters represents information content at each position. For example, if a particular nucleotide is always found at a given position, it will have the maximal height and information content, while if a position has all four nucleotides at equal frequencies, it will have a minimal height and no information content. One instance of a motif alignment is shown, which contains a variant at a high information position (boxed). The alternative allele at this position, A, results in a sequence more different from the motif represented by the PWM as measured by the motif score.

Sequence motifs are recurring patterns in genomic sequence and are frequently used to describe the sequence preferences of proteins that bind to DNA or transcribed RNA sequences. For example, TFs are proteins that are involved in gene regulation and which bind to DNA according to specific sequence preferences. Binding preferences can be represented using a position weight matrix (PWM), which summarizes alignments of experimentally bound regions and represents the probability of each nucleotide occurring at each position in the binding site. The JASPAR database [26] is the largest open-access collection of PWMs with over 200 non-redundant profiles for vertebrate TFs. Software such as the MEME suite [27] and MOODS [28] can use these matrices to scan new sequences for regions that match the PWM, typically using a certain score threshold to call a site. PWMs can be represented figuratively with sequence logos, which identify the positions of high information content in the motif (Figure 2). PWMs have also been applied in modeling splicing signals beyond the ‘essential’ two-base-pair splice sites at either end of introns (known as the splice donor and acceptor sites; Figure 1) as there are still substantial sequence preferences in the flanking regions, which serve to guide the splicing machinery.

Given that a variant is observed to overlap a particular motif, a fairly specific prediction can be made about whether the variant results in the underlying sequence being closer or further from the sequence represented by the PWM (Figure 2). Desmet *et al*. [29] describe a webserver called the Human Splicing Finder that uses PWMs to predict the effect of different alleles on splicing motifs. In addition, the Ensembl VEP can be configured to identify variants that overlap TF motifs from the JASPAR database when aligned under matched ChIP-seq peaks and computes the difference in score between the reference and alternative alleles.

A caveat with these analyses, however, is that motifs that have low information content, either because they are short or because they have relatively low sequence specificity, will align to numerous places in a genome as large as human, so further contextual evidence, such as evidence of the relevant protein binding, is important to reduce false positives. It is also the case that motif score changes and physical differences in binding affinities are not perfectly correlated, reflecting the fact that sequence motifs are an imperfect model of biophysical binding preferences [30].

### Annotation based on constraint estimated from multiple sequence alignments

Evolutionary theory predicts that deleterious variation in regions of the genome that are important for fitness will be selected against. Consequently, over evolutionary time, such regions will appear conserved compared with neutral regions. Measures of conservation can be used to identify regions of the genome where variation is expected to be more deleterious, even in the absence of specific annotations of functional elements.

Several methods have been developed to identify evolutionary conservation in both DNA and protein sequences based on aligning homologous sequences from different species. For example, the Genomic Evolutionary Rate Profiling (GERP) algorithm [31] is a widely used method for estimating constraint in genomic sequences as it can assign conservation scores to specific nucleotides, which is clearly of importance when annotating small-scale variation such as single-nucleotide variants (SNVs). GERP starts with a multiple sequence alignment (MSA) built from several species and analyses each column of the alignment independently. The number of observed substitutions is counted and then contrasted with the ‘expected’ rate, computed by considering the branch lengths of a phylogenetic tree estimated from neutral sequences to compute the neutral divergence rate. Nucleotide-resolution GERP scores can then be used to identify runs of unexpectedly constrained sequence, which can also be a useful regional annotation: these runs are defined as ‘constrained elements’. PhastCons [32], from the PHAST package, is another widely used approach to identifying conserved genomic regions and uses a phylogenetic hidden Markov model (HMM) to segment a multiple sequence alignment into conserved and non-conserved regions. Scores for individual bases in the genome can then be computed, with higher scores indicating a higher probability that the base is in a conserved element. Several other methods that can provide nucleotide-resolution conservation scores have also been developed, including phyloP [33], also from the PHAST package, and SCONE [34].

**Figure 3:**
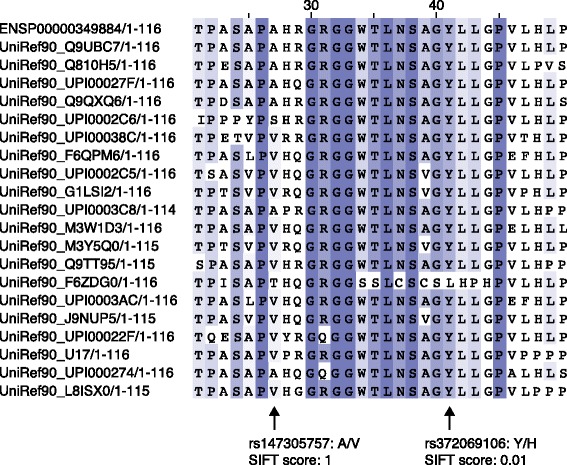
**A protein multiple alignment for the human** ***GALP*** **gene built from the SIFT alignment pipeline.** Color intensity corresponds to conservation in each column. Two variants that are predicted to alter the amino acid sequence (A/V and Y/H) are indicated by arrows and their SIFT scores are presented. Note that SIFT scores ≤0.05 are predicted to be deleterious and other scores are predicted to be tolerated.

Estimating constraint from MSA has been widely applied to predict whether a sequence variant resulting in an amino acid substitution is likely to be deleterious. The SIFT algorithm (for Sorts Intolerant From Tolerant substitutions) [35] predicts whether a substitution at a particular position in a protein sequence is expected to be deleterious for protein function. It proceeds by building a protein MSA for a given query protein sequence from closely related sequences from a database, such as UniProt [36], using a sequence-matching algorithm such as BLAST [37]. Probabilities for all possible substitutions at each position are then computed to construct a position-specific scoring matrix, where each entry in the matrix represents the probability *p*_*ca*_ of observing amino acid *a* in column *c* in the alignment. Pseudocounts, derived from a statistical model of amino acid distributions [38], are also incorporated into *p*_*ca*_ to account for the incompleteness of the sequence database used. The entries in the matrix are then normalized based on the consensus amino acid (that is, that with the maximal *p*_*ca*_) to allow a single threshold value to be used for all columns. Positions with normalized probabilities <0.05 are predicted to be deleterious (Figure 3). A recent method called FATHMM [39] also uses an MSA to identify conserved amino acid residues, but builds an HMM from the MSA and computes the differences in model probabilities between the wild-type and mutant residues to estimate the impact of the substitution. FATHMM can also incorporate ‘pathogenicity’ weights that are derived from databases of disease-implicated variants to improve predictions.

Conservation has proven to be an important signal for variant annotation, but it is blind to adaptations that have evolved since the last common ancestor of humans and the other primates. This is particularly important to consider for regulatory regions, which appear to evolve much faster than protein-coding genes. For example, Schmidt and colleagues [40] have found that most of the binding sites for the TFs they study are species-specific, even among vertebrates. Thus, while evidence that a genomic region is highly conserved can suggest that a variant might be deleterious, a lack of evidence of conservation in some specific genomic region does not necessarily imply that the region is not functional.

### Integrative approaches using supervised learning algorithms

The approaches discussed so far are based on using biological knowledge about the putative functions of particular genomic regions, or on the expectation that conserved regions are functionally important, to predict the effect of sequence variation. Rather than predefining some specific set of annotations as informative about variant consequences, an alternative approach is to attempt to learn informative annotations, or combinations of annotations, by comparing known functional variants with variants for which there is no direct evidence of functional consequences.

Several supervised machine-learning approaches have applied this methodology to the task of predicting whether a novel variant is likely to have some phenotypic consequence. The central idea is to use a ‘training set’ of variants that are categorized as either ‘functional’ or ‘benign’ to identify features, or combinations of features, that can be used to discriminate between the two classes and, hopefully, that allow the accurate classification of unseen variants.

This approach has been applied extensively in attempts to determine whether variants that are predicted to result in single amino acid substitutions (AASs), known as missense or non-synonymous variants, might be deleterious. This is an interesting class of variant as, whereas some substitutions appear to be tolerable and the underlying variants are common polymorphisms, others have been implicated in a range of genetic diseases such as cystic fibrosis, muscular dystrophy and sickle cell anemia [3]. A widely used example of this class of algorithm is PolyPhen [41], which incorporates a measure of constraint from a protein MSA (known as PSIC and somewhat similar to SIFT), along with information about the position of the substituted amino acid in a three-dimensional structure (if available), Pfam domains and other data. The algorithm trains a naïve Bayes classifier to use these features to discriminate between common polymorphic substitutions and substitutions with an annotated involvement in disease from UniProt. PolyPhen’s developers have found that the platform can discriminate between these two classes of variants with useful levels of accuracy. MutationTaster [42] uses the same naïve Bayes algorithm as PolyPhen but can also classify variants other than missense variants that can be mapped to a transcript as the algorithm incorporates a wider range of genic annotations, including conservation, splice sites and translation initiation signals.

There are also several other AAS prediction algorithms, including MutationAssessor [43], SNAP [44] and PhD-SNP [45], that take similar approaches but exploit different underlying features and training sets. Recently, a number of methods, such as Condel [46] and CAROL [47], have been developed to integrate the predictions of multiple AAS tools.

Coding regions constitute only 1 to 2% of the genome, however, and relatively little work has focused on predicting the consequences of variation in other genomic regions. A recent method called GWAVA [48] applies a similar methodology to non-coding variants. It trains a Random Forest classifier [49] to discriminate between regulatory variants that are implicated in disease from the Human Gene Mutation Database [3] and control variants from the 1000 Genomes Project [50] using a wide range of annotations relevant to gene regulation, including ENCODE project data, conservation scores and genic context.

Another recent supervised learning method that aims to identify likely functional variants across the genome is CADD [51], which incorporates both genic and regulatory annotations. Instead of learning to discriminate between known functional variants and controls, CADD uses a training set composed of variants that have become fixed in the human lineage, and which therefore presumably represent tolerable variation, and simulated variants that are not observed in human populations. This interesting approach means that, unlike the other methods discussed above, CADD can take advantage of a much larger training set and avoids ascertainment biases associated with existing databases of known disease-implicated variants.

Because these algorithms learn to identify combinations of informative annotations they can potentially identify variants acting via novel mechanisms, which rule-based approaches such as those discussed earlier would miss. However, a caveat with predictions from most machine-learning algorithms is that they cannot generally produce a human-understandable explanation of the reason for a particular prediction. Such approaches are also prone to exploit any systematic biases (such as an over-representation of variants from specific genes) in their predictions. It is therefore important to assess the performance on unseen data sets that were not used for training.

### Phenotype association techniques that can incorporate functional information

Typically, the techniques discussed above are used after an association analysis has been performed to identify potential causal variants among those linked to the association signal, or to filter variants that have been shown to segregate with disease in a pedigree study. By identifying variants that are more likely to be involved in disease *a priori*, these approaches can also potentially be used to increase the power to detect association signals in the first place. In a recent application to common disease genetics, Pickrell [52] developed an association technique called fgwas that incorporates a wide range of functional genomic annotations, and showed that the approach identifies biologically consistent enrichment of association signals in functional elements. Pickrell’s technique builds a statistical model, linking variant annotations to the probability of trait association, that is used to reweight the variants. The model gave a modest, but potentially significant, increase in power to detect associations in the 18 traits studied, which included glucose levels, height, body mass index and Crohn’s disease.

There has recently been much interest in assessing the contribution of rare variants to complex diseases, such as type 2 diabetes, arthritis and heart disease. This has prompted the development of a range of techniques to address the issue that the sample sizes required to reliably detect associations using single-locus tests are still prohibitive. One common approach to resolving this problem is to test for the association with the phenotype of a group of variants collectively rather than of each variant individually. In this context, annotations can be used to group variants according to similar biological function, such as those falling in the same gene, or to limit the work to coding variants only. SKAT [53] implements this methodology and has increased power to detect association if accurate prior ‘functionality’ weights can be assigned to the variants under consideration. The VT Test [54] is a similar method that can incorporate PolyPhen scores to up-weight probable deleterious coding variants. Experiments on both simulated and empirical data demonstrate that this approach is effective in identifying phenotypical associations with rare variants.

VAAST [55,56] is another technique that aggregates information from multiple variants to identify the genes and variants underlying genetic disease. VAAST uses information on allele frequencies in cases and controls, and combines this with AAS scores for coding variants in a likelihood framework to evaluate if a gene (or other genomic element) contributes to disease risk. VAAST also incorporates scores for non-coding variants based on a conservation metric using a general framework, which could, in principle, incorporate scores from new tools such as CADD and GWAVA. VAAST has recently been successfully applied to identify the causal coding variant for a lethal X-linked disorder [57].

## Summary

The number of variants identified in the genome has grown dramatically over the past several years, and this rich dataset has both inspired and challenged efforts to use computational techniques to functionally annotate the so-called ‘variome’. Although considerable progress is being made, in light of the limitations in the various methodologies reviewed here, we suggest that careful integration of annotations and predictions from a range of tools is a sensible strategy in practical variant-prioritization scenarios. These techniques often offer complementary information about possible functional mechanisms, and the combined results can be used to inform and generate hypotheses for subsequent validation. A further general limitation of current techniques is that they consider variants in isolation, whereas variants are not inherited independently and their genomic background might modulate any functional effects. We anticipate that techniques that can consider the combined effects of multiple variants will refine and improve predictions of variant function.

As sequencing moves from research towards clinical practice, it will become increasingly important that the variant-analysis techniques in use are validated and benchmarked for accuracy. The development of open-access databases of well-characterized variants associated with specific phenotypes will be essential. Efforts such as the Critical Assessment of Genome Interpretation (CAGI) experiment, which sets variant prediction challenges and invites predictions from all-comers, should also help to increase the accuracy and quality of predictions through collaborative competition. Technological advances in developing experimentally tractable disease models, such as induced pluripotent stem cells, and the ability to induce mutations in specific regions, for example with the CRISPR-Cas9 system [58], also offer promising opportunities to assess the performance of computational predictions.

## Abbreviations

AAS: Amino acid substitution
CAGI: Critical assessment of genome interpretation
eQTL: Expression quantitative trait locus
GERP: Genomic evolutionary rate profiling
GWAS: Genome-wide association studies
HMM: Hidden Markov model
MSA: Multiple sequence alignment
PWM: Position weight matrix
SIFT: Sorts intolerant from tolerant substitutions
SNV: Single-nucleotide variant
TF: Transcription factor
VEP: Variant effect predictor
